# Identification of prospective aging drug targets via Mendelian randomization analysis

**DOI:** 10.1111/acel.14171

**Published:** 2024-04-04

**Authors:** Rui Mao, Ji Li, Wenqin Xiao

**Affiliations:** ^1^ Department of Dermatology, Xiangya Hospital Central South University Changsha China; ^2^ Hunan Key Laboratory of Aging Biology, Xiangya Hospital Central South University Changsha China; ^3^ National Clinical Research Center for Geriatric Disorders, Xiangya Hospital Central South University Changsha China

**Keywords:** aging, Mendelian randomization, metabolite, protein

## Abstract

Aging represents a multifaceted process culminating in the deterioration of biological functions. Despite the introduction of numerous anti‐aging strategies, their therapeutic outcomes have often been less than optimal. Consequently, discovering new targets to mitigate aging effects is of critical importance. We applied Mendelian randomization (MR) to identify potential pharmacological targets against aging, drawing upon summary statistics from both the Decode and FinnGen cohorts, with further validation in an additional cohort. To address potential reverse causality, bidirectional MR analysis with Steiger filtering was utilized. Additionally, Bayesian co‐localization and phenotype scanning were implemented to investigate previous associations between genetic variants and traits. Summary‐data‐based Mendelian randomization (SMR) analysis was conducted to assess the impact of genetic variants on aging via their effects on protein expression. Additionally, mediation analysis was orchestrated to uncover potential intermediaries in these associations. Finally, we probed the systemic implications of drug‐target protein expression across diverse indications by MR‐PheWas analysis. Utilizing a Bonferroni‐corrected threshold, our MR examination identified 10 protein‐aging associations. Within this cohort of proteins, MST1, LCT, GMPR2, PSMB4, ECM1, EFEMP1, and ISLR2 appear to exacerbate aging risks, while MAX, B3GNT8, and USP8 may exert protective influences. None of these proteins displayed reverse causality except EFEMP1. Bayesian co‐localization inferred shared variants between aging and proteins such as B3GNT8 (rs11670143), ECM1 (rs61819393), and others listed. Mediator analysis pinpointed 1,5‐anhydroglucitol as a partial intermediary in the influence LCT exhibits on telomere length. Circulating proteins play a pivotal role in influencing the aging process, making them promising candidates for therapeutic intervention. The implications of these proteins in aging warrant further investigation in future clinical research.

## Graphical abstract

A conceptual framework for the identification of prospective aging drug targets. We extracted data about serum protein cis‐pQTLs from three cohorts, and outcomes from the UK Biobank and GWA/GWAS meta‐analysis. After two‐sample Mendelian randomization analysis, Bayesian co‐localization analysis, and summary‐data‐based Mendelian randomization analysis, we identified 10 age‐related proteins as the possible drug targets.

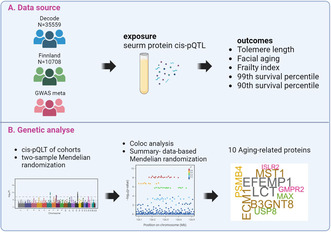

**Abbreviations**
FA: facial aging
FI: frailty index
GWAS: genome‐wide association studies
IV: instrumental variables
MR: mendelian randomizatio
MR‐IVW: inverse variance‐weighted MR
pQTL: protein quantitative trait loci
QTL: quantitative trait locus
SMR: Summary‐data‐based Mendelian randomization
STROBE‐MR: strengthening the reporting of observational studies in epidemiology‐mendelian randomisation
TL: telomere length
T2DM: type 2 diabetes mellitus

## 1 INTRODUCTION

Aging is a process with the accumulation of aging cells, the decline of normal physiological functions, and the increasing risk of aging‐related diseases and death (Cătană et al., 2018). There are a series of biomarkers that can evaluate the physiological aging process from microcosmic to macroscopic level, such as telomere length (Ferkingstad et al., 2021), facial aging (FA), and frailty index (FI) (Bao et al., 2023). Due to the fact that aging has become one of the biggest risk factors of chronic diseases, identification and manipulating aging may improve these aging‐related diseases, prevent premature death, and extend lifespan (Guo et al., 2022).

Over the past decades, many efforts have been made to find anti‐aging genes and substances. However, among these thousands of compounds, only a few was proved to be effective. Besides, these interventions which target single molecular have significant deficiency due to neglecting the complexity for living systems (Rybina et al., 2023). Therefore, developing novel strategies to combat aging is still imminent.

Human proteins participant in multiple biological activities and are served as a major part of medical targets (Zheng et al., 2020). Therefore, targeting certain proteins to prevent aging may have great feasibility. Mendelian randomization is an approach to evaluate the causality between exposure and outcome by using single nucleotide polymorphisms (SNPs) from genome‐wide association studies (GWAS) as genetic instruments (Sekula et al., 2016). MR analysis has been widely used to discover novel drug target, however, here lacks MR research about integrating GWAS and quantitative trait locus (QTL) data to explore anti‐aging therapy (Swerdlow et al., 2012).

In this study, we aimed to find critical proteins which could regulate aging. First of all, we proved the causal effects of proteins had on aging proxy indicators by MR analysis and strengthened these results by another validation cohort. Subsequently, reverse causality detection, Bayesian co‐localization analysis, phenotype scanning, and SMR analysis further verified our conclusion. Finally, we investigated the mediation effect of metabolic factors on the association between the candidate protein and aging.

## 2 MATERIALS AND METHODS

### 2.1 Data availability

**FIGURE 1:**
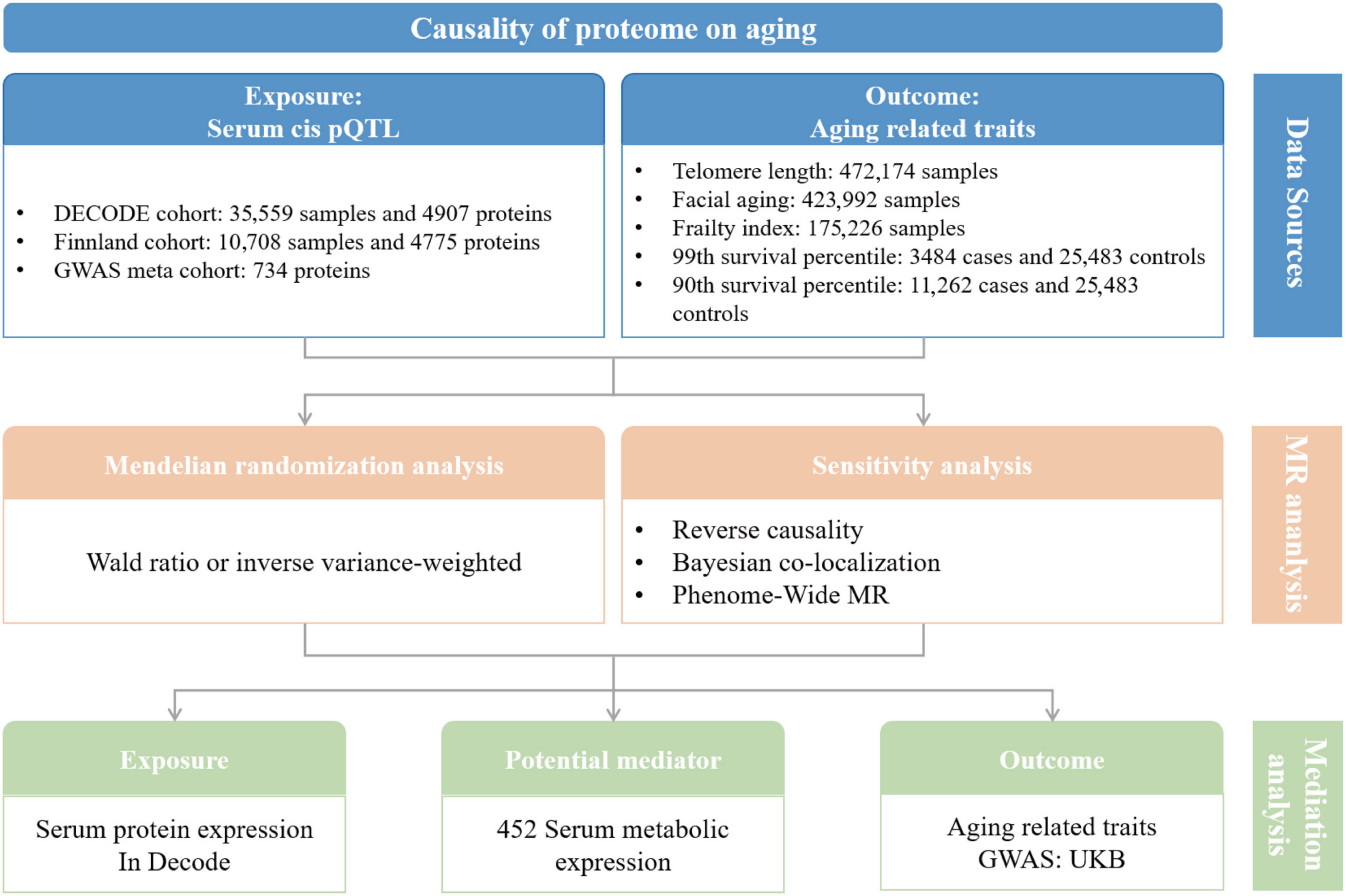
A schematic representation detailing the comprehensive research framework and procedural workflow adopted in this study.

This study meticulously complies with the “Strengthening the Reporting of Observational Studies in Epidemiology‐Mendelian Randomisation (STROBE‐MR)” guidelines, as detailed in eTable 1 in Appendix S1 (Skrivankova et al., 2021). Data derived from genome‐wide association studies (GWAS) and pQTL were obtained solely from open‐access repositories that have secured ethical approval. An exhaustive account of all datasets employed is available in eTable 2 in Appendix S1. Figure 1 illustrates the unified analytical approach undertaken in this research.

### 2.2 Plasma protein quantitative trait loci datasets

We sourced summary‐level genetic association data on 4907 circulating proteins from an extensive pQTL study involving 35,559 Icelandic participants, known as the DECODE cohort (Ferkingstad et al., 2021). This proteomic analysis employed the multiplexed, modified aptamer‐based binding assay, specifically the SOMAscan version 4. After controlling for confounding variables like age and sex, we subjected protein levels to rank‐inverse normal transformation. The resulting standardized residuals were harnessed as phenotypes in genome‐wide association analyses, conducted using the BOLT‐LMM linear mixed model approach. Comprehensive details regarding the GWAS procedures can be found in the primary reference (Ferkingstad et al., 2021).

For this study, we instituted stringent inclusion criteria for pQTLs:

- Achieved genome‐wide significance in their associations (*p* < 5 × 10^−8^);
- Positioned externally to the major histocompatibility complex (MHC) region (chr6, 26–34 Mb);
- Demonstrated an unequivocal independent association, adhering to an LD clumping *r* ^2^ threshold of <0.001;
- Classified as a cis‐acting pQTL.

To validate our results, we consulted plasma pQTL datasets from contemporary literature. Pietzner et al. (2021) analyzed 4775 plasma proteins in a cohort of 10,708 Finnish participants, whereas Zheng et al. (2020) amalgamated data from five preceding GWAS (Emilsson et al., 2018; Folkersen et al., 2017; Suhre et al., 2017; Sun et al., 2018; Yao et al., 2018), aggregating 738 cis‐acting SNPs tied to 734 proteins, designated as the meta‐cohort.

### 2.3 Outcome data sources

Genetic variants linked to TL and FA were sourced from the UK Biobank, which encompassed sample sizes of 472,174 individuals (average age 56.1 ± 7.9) and 423,999 individuals (aged between 40 and 69), respectively (Codd et al., 2021; Roberts et al., 2020). Within the UK Biobank, mean leukocyte TL was gauged in a mixed leukocyte population via the multiplex quantitative polymerase chain reaction (qPCR) method. This technique represented TL as the T/S ratio (telomeric repeats to single‐copy genes). For FA evaluation, participant perceptions about their age were coded: 1 for appearing younger, 0 for seeming older, and 0.5 for resembling their age. These observations, made by independent third parties, underpinned the determination of participants' FA based on perceived versus actual age. Through the mixed linear model analysis, FA emerged as an ordered categorical variable. Statistical data, originally on linear scales, transitioned to log‐odd ratios (OR) via the Taylor extended series, with OR >1 signifying a higher likelihood of appearing younger. Significant genetic variants associated with FI were sourced from the GWA meta‐analysis, encompassing 164,610 UK Biobank participants (average age 64.1 ± 2.8) and 10,616 TwinGene participants (average age 58.3 ± 7.9; Atkins et al., 2021). Adherence to defects was denoted by an Atkins A score of 0 or 1 (0 indicating none). Thus, an individual's FI equated to the defect count over the aggregate of 49 defects as described in the preceding study (eTable 2 in Appendix S1). A higher FI value denoted heightened individual frailty, with UK Biobank and TwinGene participants showcasing average defect proportions of 0.129 ± 0.075 and 0.121 ± 0.080, respectively. Genetic variants tied to longevity emanated from a GWAS meta‐analyses, inclusive of 11,262/3484 cases surpassing the 90th/99th survival percentile and 25,483 controls. In this analysis, cases represented individuals exceeding the 90th percentile, based on life table data from pertinent countries, gender, and birth cohorts. Controls were demarcated as those who passed away on or before the 60th percentile or reached that age at the last follow‐up (Deelen et al., 2019).

### 2.4 Mendelian randomization analysis

In this study, circulating proteins were selected as exposure, and aging proxy indicators (TL, FA, FI, and 90th/99th survival percentile) were selected as the outcome. MR was performed with “TwoSampleMR.” In instances where a single pQTL was available for a protein, we utilized the Wald ratio. However, when multiple genetic instruments were at our disposal, we applied the inverse variance‐weighted MR (MR‐IVW) (Deng et al., 2022), subsequently followed by a heterogeneity analysis. The odds ratio (OR) for the augmented risk of aging‐related traits is denoted by the increase in the standard deviation (SD) of plasma protein levels.

To deem an instrumental variable valid, it must satisfy three fundamental assumptions, as illustrated in eFigure 1 in Appendix S2.

#### 2.4.1 Association with exposure

The genetic variation must have a strong association with the exposure. This association was verified by calculating the instrument's *F*‐statistic, employing the formula: squared beta divided by the squared standard error (Burgess & Thompson, 2011). To mitigate weak instrument bias, we sought an *F*‐statistic exceeding 10. The MR‐power online tool (Burgess, 2014) was instrumental in determining the robustness of our MR estimates.

#### 2.4.2 Independence from confounders

The selected genetic variants should function independently of confounders that potentially influence the exposure‐outcome nexus. Leveraging the PhenoScanner database (Kamat et al., 2019), we identified traits—excluding the exposure—with significant ties to outcomes (TL, FA, and FI) at thresholds of *p* < 5 × 10^−8^. Literature review pinpointed traits such as depression, type 2 diabetes mellitus (T2DM), and ischemic stroke as potential risk factors for outcomes. To minimize horizontal pleiotropy, we precluded variants associated with these particular traits.

#### 2.4.3 Exclusive influence on the outcome through exposure

The genetic variants should impact the outcome strictly via the determined exposure. To counteract confounding from inherent population stratifications, we maintained methodological consistency by centering our analysis on a homogenous ancestry group. Additionally, linkage disequilibrium poses confounding risks. This motivated our adoption of a Bayesian co‐localization analysis, designed to ascertain the likelihood of genetic confounding (Giambartolomei et al., 2014).

### 2.5 Reverse causality reaction

Utilizing the established criteria, we embarked on a bidirectional MR analysis to unearth potential reverse causality (Davey Smith & Hemani, 2014). Multiple methods—MR‐IVW, MR‐Egger, and the weighted median approach—were employed to quantify the effects. A *p*‐value threshold of less than 0.05 was set as the criterion for statistical significance.

### 2.6 Bayesian co‐localization analysis

We conducted a Bayesian co‐localization analysis to ascertain the likelihood that two traits are influenced by the same causal genetic variants. This was assessed using the “coloc” package available at https://github.com/chr1swallace/coloc. The analysis yielded posterior probabilities for five hypotheses concerning the potential shared genetic variant between the proteome and aging (Folkersen et al., 2017). In our investigation, we specifically evaluated the posterior probability of hypothesis 3 (PPH3) and hypothesis 4 (PPH4). Under PPH3, the protein and aging are influenced in the region by distinct variants. Conversely, PPH4 posits that both the protein and aging‐related traits are associated with the region due to shared genetic variants. For co‐localization evidence, we used coloc.abf and coloc.susie algorithms. A gene was deemed to exhibit co‐localization if it presented a gene‐based PPH4 exceeding 80%, as validated by at least one of the algorithms (Burgess et al., 2019; Deng et al., 2022).

### 2.7 Summary‐data‐based Mendelian randomization (SMR) analysis and heterogeneity in dependent instruments (HEIDI) test

When utilizing protein Quantitative Trait Loci (pQTL) as a research tool, effect estimates are derived via SMR method, which leverages aggregate‐level data from GWAS and pQTL studies to explore the associations between protein expression levels and specific outcomes of interest (Zhu et al., 2016). Allele coordination and analysis were conducted using the SMR software, version 1.3.1, accessible at (https://yanglab.westlake.edu.cn/software/smr/). An inverse variance‐weighted Mendelian randomization (IVW‐MR) approach was utilized to integrate the effect estimates. Within this SMR framework, HEIDI test was applied to ascertain if the observed gene expression‐outcome associations were attributable to a linkage scenario. This analysis was facilitated by the SMR software (Zhu et al., 2016), where a HEIDI test yielding a *p*‐value less than 0.01 indicates that the association might stem from linkage (Chauquet et al., 2021). Given that a single SNP may influence the expression of multiple genes, thereby introducing the risk of horizontal pleiotropy, we scrutinized other genes within a 1 Mb window whose expression was significantly linked to genetic tool variation. Subsequently, SMR analysis was conducted to assess whether the expression of these genes correlated with age‐related traits, thereby evaluating the potential impact of horizontal pleiotropy on the findings.

### 2.8 Mediation analysis

The purpose of the mediation analysis was to elucidate the metabolites (mediators) that underlie the relationships between proteins and aging proxy indicators.

#### 2.8.1 SNP identification and screening

Initially, we identified significant SNPs associated with the exposure from the GWAS. Subsequently, we excluded SNPs in linkage disequilibrium and isolated the residual SNPs from the GWAS corresponding to the mediating variable.

#### 2.8.2 Causal effect assessment

We estimated the causal influence of the exposure on the mediators, denoted as BetaEM. Using a congruent approach, the causal impact of the mediators on outcomes, BetaMO, was determined. The overall causal effect of exposure on outcomes was represented as BetaEO.

#### 2.8.3 Causal significance

Should BetaEM, BetaMO, and BetaEO all be significant, it would suggest a potential causal relationship between the exposure and outcome, possibly mediated by the identified mediators.

#### 2.8.4 Mediation effect calculation

We computed the indirect relationship, represented by BetaEM * BetaMO, as the mediation effect (Burgess et al., 2015). The proportion of this mediation effect is articulated by the equation (BetaEM * BetaMO)/BetaEO.

#### 2.8.5 Error estimation

For these indirect effects, standard errors were calculated utilizing the delta method (Sanderson, 2021).

### 2.9 Phenome‐wide MR analysis

To explore potential side effects linked to our therapeutic target gene, we conducted phenome‐wide MR analyses. Utilizing pQTL data for the aging‐target protein as the exposure and contrasting this with aggregated genome‐wide association study (GWAS) data on various diseases from the UK Biobank cohort (*n* = 408,961) as the outcome, we aimed to identify unforeseen consequences. Lee et al.'s comprehensive GWAS analyses within the UK Biobank, employing the SAIGE (V.0.29) methodology—an advanced technique for the effective use of generalized mixed models to manage imbalanced case–control ratios—provided a robust foundation for our investigation (Zhou et al., 2022). Our analysis encompassed 1402 traits, representing distinct diseases, each with at least 50 case instances, carefully excluding traits directly related to aging or its associated conditions to avoid confounding effects. Disease‐associated SNP statistics were retrieved from the SAIGE GWAS database (https://www.leelabsg.org/resources), with detailed methodologies outlined in their respective publication. The Wald ratio method was applied for MR analysis to maintain consistency in estimating causal relationships, with a stringent significance threshold of *p* < 0.05/1402 applied to identify significant associations.

### 2.10 Cell culture and treatment

Human dermal fibroblasts (HDF) were obtained from NTCC and were cultured in DMEM supplemented with 10% fetal bovine serum, penicillin–streptomycin. For UV‐irradiation treatment, cells were treated with a total energy of 10 J/cm^2^ UV irradiation by UV source.

### 2.11 Lentiviral shRNAs and plasmid

shRNAs cloned into the third‐generation pLKO.1‐puro vector were purchased from Sigma‐Aldrich. Lentivirus packaging and testing were performed as previously described (Deng et al., 2021). HDFs for subsequent treatment were infected with lentiviruses in growth medium containing 8 μg/mL polybrene and selected in 2 μg/mL puromycin for 7 days.

### 2.12 RT–qPCR

Total RNA was extracted from HDFs using TRIzol Reagent (Thermo Fisher Scientific). mRNA was reverse‐transcribed using the Maxima H Minus First Strand cDNA Synthesis Kit with dsDNase (Thermo Fisher Scientific) based on its instructions. qPCR was conducted with iTaqTM Universal SYBRGreen Supermix (Bio‐Rad) on a LightCycler 96 (Roche) thermocycler.

### 2.13 Relative telomere length measurement

Mean HDF TL was measured in a mixed HDF population by the multiplex quantitative polymerase chain reaction (qPCR) method following the manufacturer's instructions (Gene Pool, China). The relative TL was assessed by the T/S ratio (telomeric repeats to single‐copy genes).

### 2.14 Senescence‐associated β‐galactosidase (SA‐β‐gal) assay

SA‐β‐gal staining of HDFs was performed by an SA‐β‐gal staining kit (Beyotime Biotechnology, China) according to the manufacturer's instruction. Briefly, cells were fixed in stationary liquid for 5 min. After washing with PBS three times, samples were incubated in SA‐β‐gal solution at 37°C for 16 h. Enzymatic reaction was stopped by PBS. Five images were taken from random fields in each group using microscope. The ratio of positive cells was determined by counting the blue cells and dividing by the total number of observed cells.

### 2.15 Statistical analysis

To correct for multiple comparisons, we adjusted *p*‐values using the Bonferroni correction, setting a significance threshold at *p* < 0.05/*n*, where “*n*” represents the effective number of exposure or outcome variables. For isolated analyses performed throughout this research, a *p*‐value threshold of less than 0.05 was established to indicate statistical significance. All computational and statistical evaluations were carried out using the R software (version 4.2.2). The incorporated packages included “TwoSampleMR (version 0.5.6),” “coloc (version 5.1.0.1),” “phenoscanner (version 1.2.2),” “Rmediation (version 1.0),” and “CMplot (version 4.3.1).”

## 3 RESULTS

### 3.1 Exploring aging‐related drug targets

**FIGURE 2:**
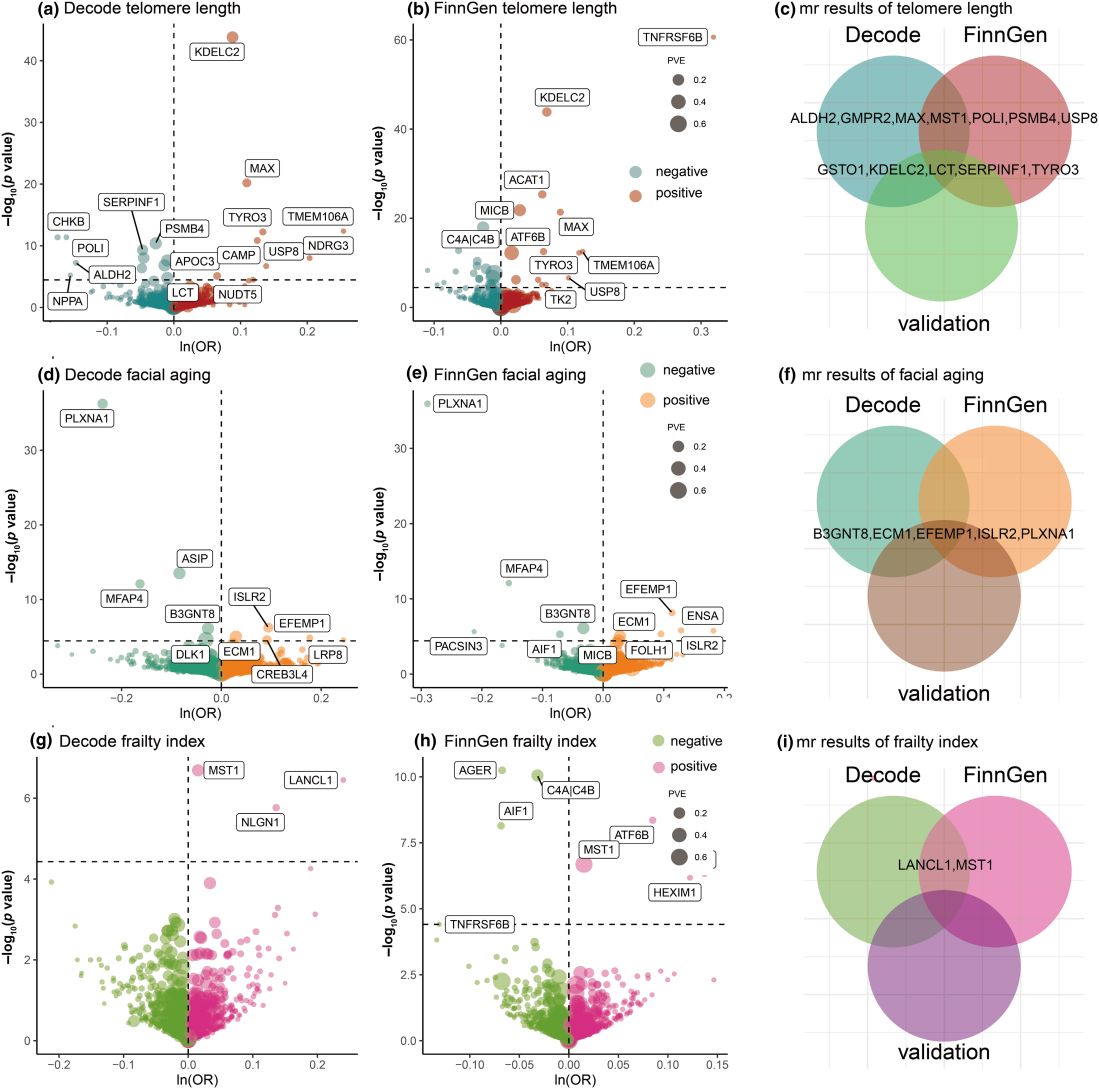
Process of Mendelian randomization (MR) analysis for identifying potential drug targets of aging across three distinct cohorts. (a) Volcano gram illustrating the risk association between plasma proteins of the DECODE cohort and TL. A delineated horizontal black line signifies *p* = 3.41 × 10^−5^ (0.05/1465). (b) Volcano gram representing the risk association of plasma proteins from the Finnland cohort with TL, marked by a dashed horizontal black line at *p* = 3.42 × 10^−5^ (0.05/1460). (c) Comparative analysis intersecting drug targets for TL from the three cohorts. In both the training and replication cohorts, seven proteins, such as MST1, GMPR2, MAX, POLI, PSMB4, USP8, and ALDH2, attained significance after adjustment. Despite their absence in the validation cohort, they were deemed noteworthy. (d) Volcano gram showcasing the risk association between plasma proteins from the DECODE cohort and FA, indicated by a delineated horizontal black line at *p* = 3.56 × 10^−5^ (0.05/1401). (e) Volcano gram highlighting the risk association of plasma proteins from the Finnland cohort with FA, with a dashed horizontal black line at *p* = 3.78 × 10^−5^ (0.05/1321). (f) Comparative intersection of drug targets for FA sourced from the three cohorts. (g) Volcano gram demonstrating the risk association between plasma proteins from the DECODE cohort and FI, highlighted by a delineated horizontal black line at *p* = 3.72 × 10^−5^ (0.05/1343). (h) Volcano gram indicating the risk association of plasma proteins from the Finnland cohort with FI, marked by a dashed horizontal black line at *p* = 3.90 × 10^−5^ (0.05/1279). (i) Intersection and comparative analysis of drug targets for FI as derived from the three cohorts. In both the training and replication cohorts, MST1 and LANCL1 attained significance after adjustment. Despite their absence in the validation cohort, they were deemed noteworthy. The odds ratio (OR) for increased aging risk is depicted as a standard deviation (Ferkingstad et al., 2021) increment for each rise in plasma protein level. ln, natural logarithm; PVE, proportion of variance explained.

Through meticulous screening, we identified 1858 cis‐pQTLs corresponding to 1553 proteins in the DECODE cohort, 1739 cis‐pQTLs for 1561 proteins in the Finnland cohort, and 738 cis‐pQTLs associated with 734 proteins in the meta‐cohort. Detailed insights are provided in eTables 3–5 in Appendix S1. For methodical analysis, the DECODE, Finnland, and meta‐cohorts were, respectively, categorized as exposure datasets for training, replication, and validation stages. Upon conducting a MR analysis with a stringent Bonferroni correction threshold, we identified several candidate proteins for aging in the three cohorts (Table 1 and eTables 6–8 in Appendix S1). To be specific, KDEL motif containing 2 (KDELC2), tyrosine‐protein kinase receptor (TYRO3), MYC‐associated factor X (MAX), and ubiquitin‐specific peptidase 8 (USP8) presented a positive correlation with TL, whereas glutathione S‐transferase omega 1 (GSTO1), lactase (LCT), serpin family F member 1 (SERPINF1), aldehyde dehydrogenase 2 family member (ALDH2), DNA polymerase iota (POLI), macrophage stimulating 1 (MST1), guanosine monophosphate reductase 2 (GMPR2), and proteasome 20S subunit beta 4 (PSMB4) had a negative correlation (Figure 2a–c). Furthermore, extracellular matrix protein 1 (ECM1), EGF containing fibulin extracellular matrix protein 1 (EFEMP1), and immunoglobulin superfamily containing leucine rich repeat 2 (ISLR2) may promote FA while UDP‐GlcNAc:betaGal beta‐1,3‐*N*‐acetylglucosaminyltransferase 8 (B3GNT8) and plexin A1 (PLXNA1) may prevent it (Figure 2d–f). As for FI, LanC like glutathione S‐transferase 1 (LANCL1) and macrophage stimulating 1 (MST1) were associated with increased FI only in Decode and FinnGen cohorts (Figure 2g–i). MR analyses showed that there were significant causal associations of proteome on longevity (90th survival percentile) (eFigure 2 in Appendix S2).

**TABLE 1:** MR results for proteins significantly associated with aging after Bonferroni correction in three cohorts.

Outcome	Variable	Decode		Finnland		Meta‐cohort		Steiger_*p*val
		Beta	*p*‐Value	Beta	*p*‐Value	Beta	*p*‐Value	
TL	GSTO1	−0.01	1.48E‐07	−0.01	1.48E‐07	−0.01	2.12E‐05	2.68E‐275
	KDELC2	0.09	1.48E‐44	0.07	1.48E‐44	0.07	1.48E‐44	7.67E‐205
	LCT	−0.01	1.61E‐05	−0.01	1.61E‐05	−0.01	1.61E‐05	3.73E‐279
	SERPINF1	−0.05	4.83E‐10	−0.04	4.83E‐10	−0.03	4.83E‐10	5.79E‐194
	TYRO3	0.13	5.70E‐13	0.12	5.70E‐13	0.11	2.88E‐15	2.03E‐30
	ALDH2	−0.15	5.93E‐8	−0.07	8.56E‐9	—	—	1.49E‐14
	POLI	−0.16	4.13E‐12	−0.06	1.71E‐13	—	—	1.34E‐10
	MST1	−0.01	2.41E‐8	−0.01	2.29E‐8	—	—	5.61E‐274
	GMPR2	−0.05	4.41E‐7	−0.03	4.41E‐7	—	—	2.88E‐155
	MAX	0.11	6.32E‐21	0.09	4.27E‐22	—	—	2.43E‐77
	PSMB4	−0.03	3.93E‐11	−0.04	4.28E‐11	—	—	8.14E‐270
	USP8	0.14	1.94E‐7	0.10	2.34E‐7	—	—	2.69E‐14
FA	B3GNT8	−0.03	7.88E‐07	−0.03	7.96E‐07	−0.03	7.88E‐07	2.55E‐273
	ECM1	0.03	9.84E‐06	0.03	9.84E‐06	0.02	1.09E‐05	1.56E‐275
	EFEMP1	0.18	1.40E‐05	0.11	7.01E‐09	0.13	7.01E‐09	2.84E‐25
	ISLR2	0.09	5.91E‐07	0.13	1.58E‐06	0.11	1.80E‐06	7.21E‐130
	PLXNA1	−0.24	5.75E‐37	−0.29	1.17E‐36	−0.31	1.67E‐33	2.03E‐127
FI	LANCL1	0.24	3.56E‐07	0.14	4.90E‐07	—	—	2.34E‐08
	MST1	0.02	2.05E‐07	0.02	2.05E‐07	—	—	1.54E‐234

### 3.2 Sensitivity analysis

**FIGURE 3:**
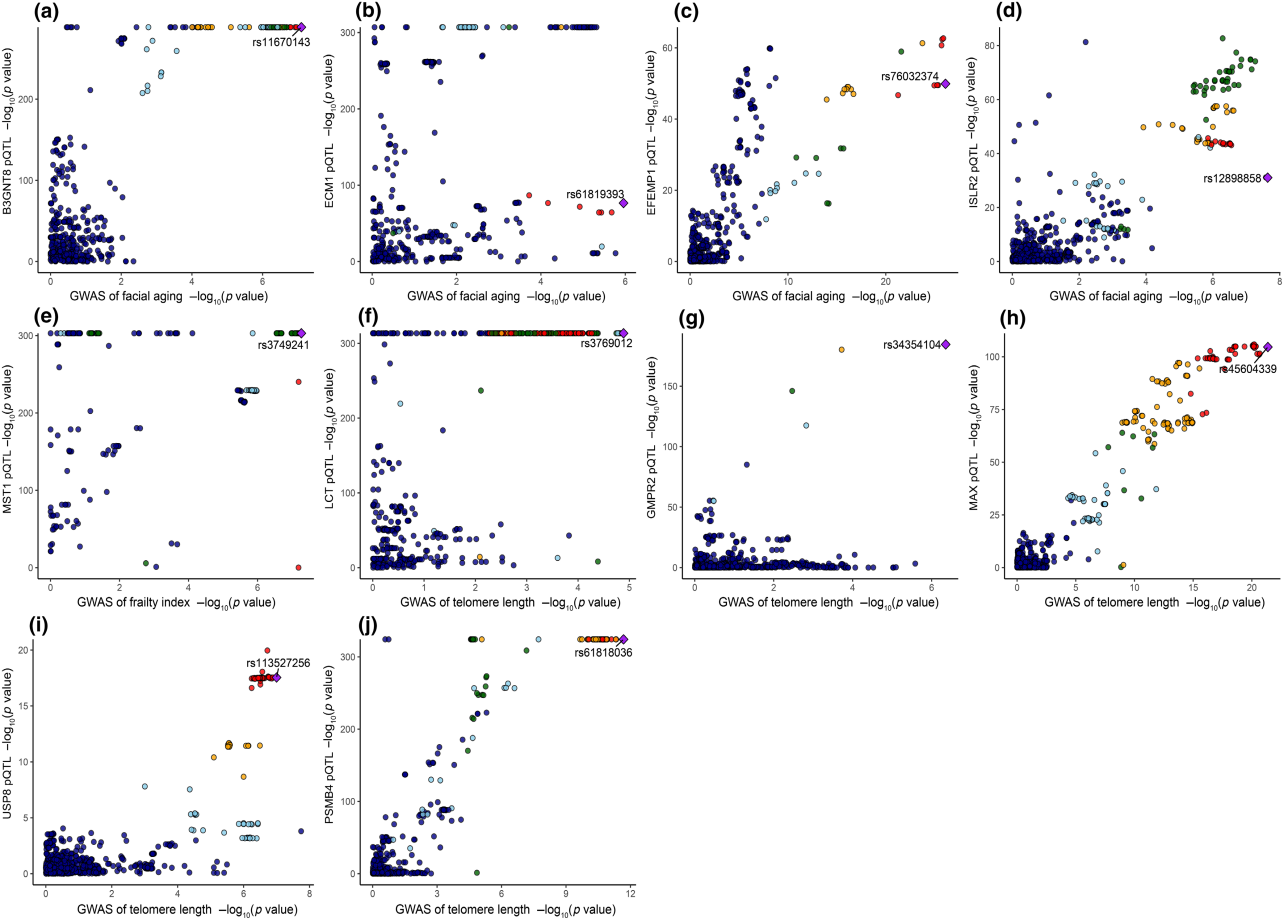
Bayesian co‐localization analysis assessing potential causal relationships between select plasma proteins and aging. Co‐localization analysis pertaining to the B3GNT8 (a), ECM1 (b), EFEMP1 (c), ISLR2 (d) plasma protein and FA. Co‐localization analysis pertaining to the MST1 (e) plasma protein and FI. LCT (f), GMPR2 (g), MAX (h), USP8 (i), and PSMB4 (j) plasma protein and FA. Diamond‐shaped purple markers highlight the single nucleotide polymorphisms (SNPs) possessing the smallest combined *p*‐value from both the corresponding protein GWAS and the GWAS of aging‐related trait.

Cochran's IVW Q test indicated that the instrumental variables (IVs) exhibited no significant heterogeneity in our primary analysis, as detailed in eTable 6 in Appendix S1. Additionally, the MR‐Egger intercepts from the primary analysis revealed no indications of pleiotropy (eTable 6 in Appendix S1). We also employed Bayesian co‐localization to validate the likelihood of shared causal genetic variants between aging traits and protein expressions. The findings pinpointed 11 proteins, notably B3GNT8 (rs11670143), ECM1 (rs61819393), EFEMP1 (rs76032374), ISLR2 (rs12898858), MST1 (rs3749241), LCT (rs3769012), GMPR2 (rs34354104), MAX (rs45604339), USP8 (rs113527256), and PSMB4 (rs61818036), that shared consistent genetic variants (see Figure 3 and eTable 9 in Appendix S1; PP.H4.abf > 0.8). Bidirectional MR analysis suggested no causal effect of aging on the identified proteins, with the exception of GMPR2 (refer to eTable 10 in Appendix S1). The application of Steiger filtering further corroborated this directional association, as seen in Table 1. A comprehensive phenotypic examination revealed no associations between these 10 proteins and depression, T2DM, ischemic stroke, or other established aging‐related risk factors (eTable 11 in Appendix S1). The SMR analysis suggested that the expression levels of the 10 proteins are significantly causally related to aging‐related phenotypes. Meanwhile, the HEIDI test indicated no evidence of horizontal pleiotropy for 9 out of the 10 proteins, except MST1 (eTable 12 in Appendix S1). In assessing the risk of horizontal pleiotropy for MST1, we identified genes near MST1 whose expression was significantly related to genetic instrumental variants. Subsequent SMR analysis showed that only MST1, and not these nearby genes, had a significant causal relationship with aging‐related phenotypes (eTable 13 in Appendix S1).

### 3.3 Phenome‐wide MR analysis

Considering that many drugs exert their effects via the bloodstream, we investigated the potential impact of the 10 blood protein expressions on other health conditions. An extensive MR analysis was conducted on 1402 conditions and traits from the UK Biobank, detailed in eTables 18–27 in Appendix S1. Our findings indicated that ECM1 is linked to osteoarthrosis and other arthropathies; EFEMP1 is associated with conditions such as inguinal hernia and diverticulitis, ISLR2 with abdominal hernia, MST1 with hypovolemia and heartburn, USP8 with unspecified monoarthritis, and PSMB4 with hypothyroidism (refer to eFigures 3 and 4 in Appendix S2; *p* < 0.05/1402 = 3.57e^−5^).

### 3.4 Mediation MR analysis

**FIGURE 4:**
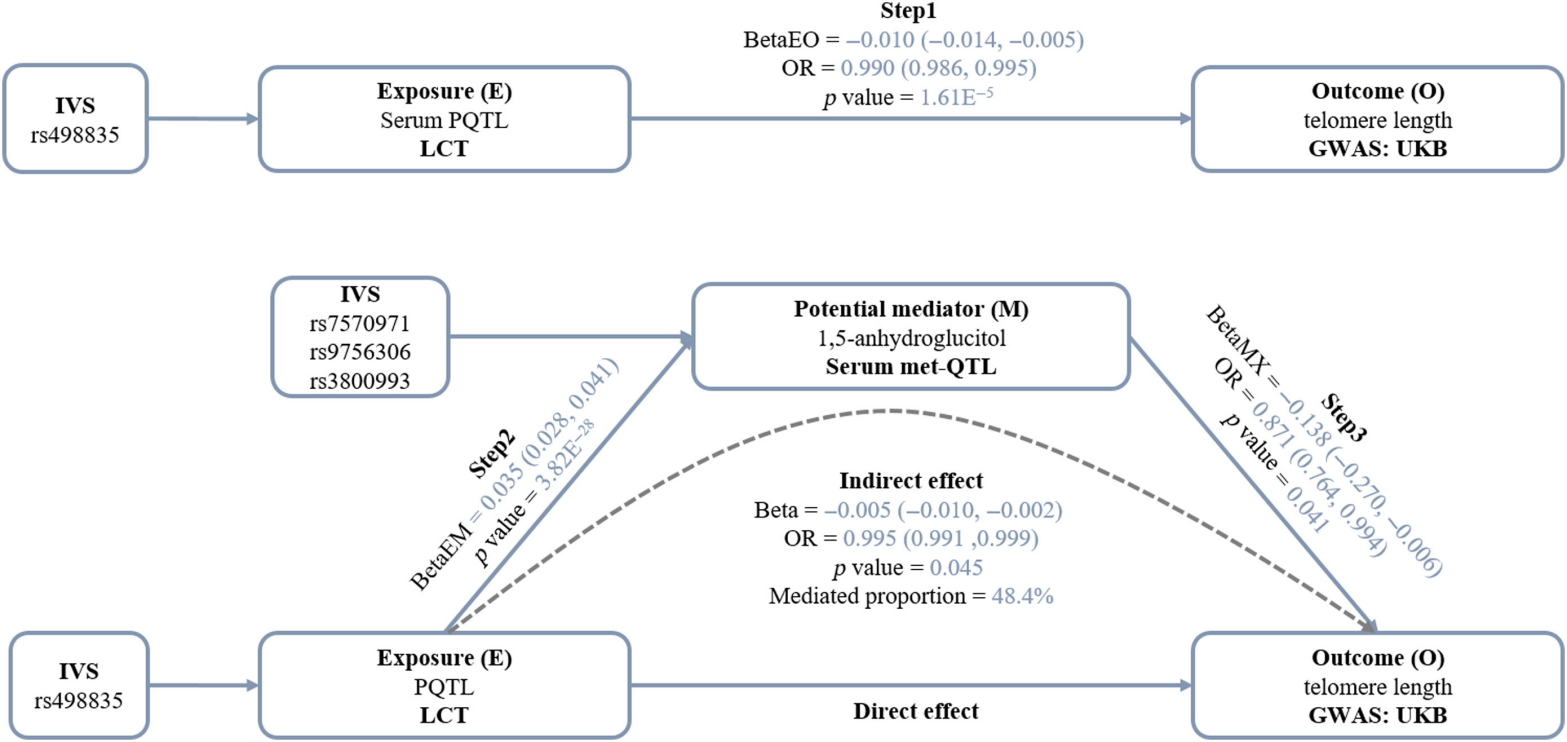
An analytical depiction of the mediation impact of LCT on TL via circulating blood metabolites executed using a two‐step Mendelian randomization approach. The term “Direct effect” refers to the influence of LCT on TL after adjusting for mediators. Conversely, “Indirect effect” denotes the influence exerted by LCT on TL through the intermediary mediator. GWAS, genome‐wide association study.

**FIGURE 5:**
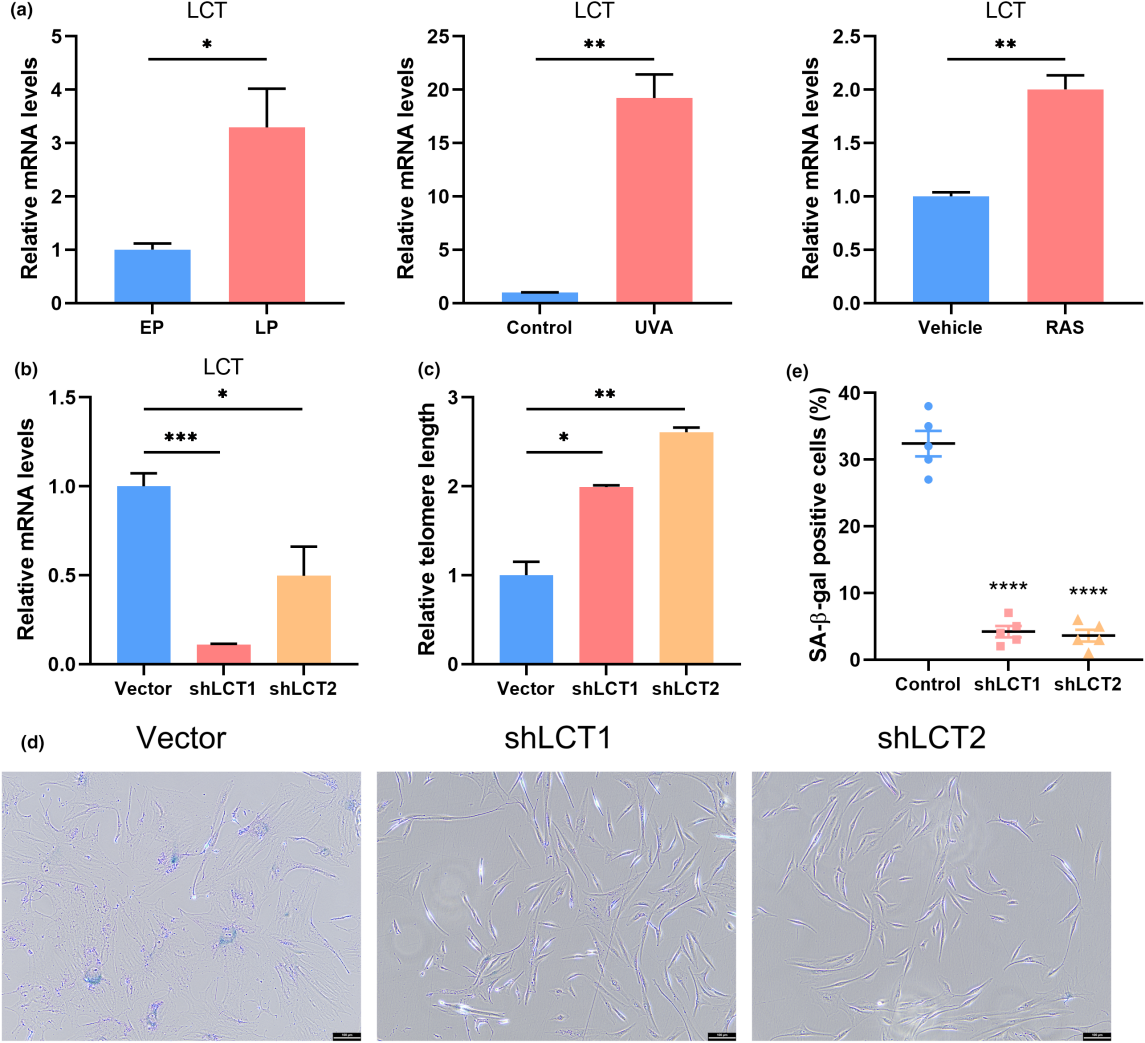
Knockdown the expression of LCT prevents aging in HDFs. (a) The mRNA expression levels of LCT in HDFs (*n* = 3 for each group). EP, early passage; LP, late passage. (b) The mRNA expression levels of LCT in HDFs carrying either a vector expressing a scramble shRNA or LCT shRNA (*n* = 3 for each group). (c) The relative telomere length of HDFs carrying either a vector expressing a scramble shRNA or LCT shRNA (*n* = 3 for each group). (d) Representative images and quantification of SA‐β‐gal–positive HDFs treated as indicated. Scale bar, 100 μm. (e) Quantification of SA‐β‐gal–positive HDFs in (d) (*n* = 5 for each group). All results are representative of at least three independent experiments. Data represent the mean ± SEM. *p < 0.05, **p < 0.01, and ***p < 0.001. Student's *t*‐test was used.

Aging is commonly associated with metabolic alterations (van der Spek et al., 2020). Consequently, we hypothesized that protein expression might modulate the transportation or synthesis of metabolites, altering the concentration of circulatory small molecules, ultimately culminating in senescence. The serum cis‐metQTL data, sourced from a GWAS, investigated genetic underpinnings of 452 circulating metabolic traits, determined using chromatography techniques in 7824 adults from European cohorts (Shin et al., 2014). Our primary analysis leveraged the genetic instrumentation of the protein to assess its causal influence on potential mediators. Among 452 candidates, we identified robust causal links between LCT and 1,5‐AG and X‐12696, and between LANCL1 and Citrulline (eTable 14 in Appendix S1, *p* < 9.21e‐6). In the subsequent phase, we explored the causal effects of these mediators on aging trait susceptibility using the genetic instruments of the three metabolites (eTable 15 in Appendix S1). This highlighted significant causal effects of 1,5‐AG (eTable 16 in Appendix S1). Crucially, exposure and mediated instrumental variables were distinct, with independent SNP associations maintained in our two‐step MR analysis. Indirectly, LCT's impact on TL, channeled through 1,5‐AG concentration, equated to −0.005 (95% CI, −0.010 to 0.002; *p* = 0.045), explaining 48.4% of the mediation (Figure 4 and eTable 17 in Appendix S1). To prove this result, we evaluated the expression of LCT in HDFs and found higher mRNA level of LCT in senescent cells induced by replication, UVA and Ras. Furthermore, after knockdown the expression of LCT by shRNA, the telomere length of HDFs increased and the number of SA‐β‐gal–positive HDFs was significantly reduced, which proved that inhibiting the expression LCT may delay aging processes (Figure 5).

## 4 DISCUSSION

In our investigation, we employed two‐sample MR analysis, Bayesian co‐localization, and SMR analysis to examine the associations between proteins and aging. Our findings suggest that seven proteins—MST1, LCT, GMPR2, PSMB4, ECM1, EFEMP1, and ISLR2—may contribute to accelerated aging, as evidenced by decreased telomere length (Ferkingstad et al., 2021), increased frailty index (FI), and augmented facial aging (FA) across three cohorts. Conversely, four proteins—MAX, B3GNT8, and USP8—emerged as potential targets for decelerating the aging process and enhancing lifespan. Moreover, our analysis indicates that 1,5‐anhydroglucitol could serve as a mediator in the relationship between LCT and aging. In these pro‐aging proteins, LCT participates in digesting lactose and is uniquely expressed in small intestine (Labrie et al., 2016). The expression and activity of LCT declined in elderly mammals (Labrie et al., 2016; Lee et al., 1997; Yazawa et al., 1981), which is inconsistent with our results. This phenomenon may be attributed to the species specificity and tissue specificity of protein expression. The impact LCT had on TL may be mediated by 1,5‐anhydroglucitol according to our mediation analysis. 1,5‐Anhydroglucitol is a glycemic marker which is competitive to glucose for reabsorption, and its level of older human is lower than younger individuals (Dungan, 2008). We assumed that the over‐activation of LCT increased the expression of 1,5‐anhydroglucitol by glucose metabolic disorders, leading to bad glucometabolic control and finally promoting aging (Chaleckis et al., 2016; Funasako et al., 1994; Garvey et al., 2014).

PSMB4 is a member of the ubiquitin‐proteasome family and is the first proteasome subunit that found to possess oncogenic properties (Wang et al., 2018). PSMB4 facilitates cancer cell survival and tumor growth and is associated with poor prognosis in various tumor types, such as breast, lung, skin, and ovary (Ali et al., 2022). The underlying relationship between tumor promoting and aging accelerating effects of PSMB4 calls for further exploration.

Skin aging is the most recognizable outcome of aging, which also directly reflects the degree of whole‐body aging to some extent (Purba et al., 2001). ECM1 is a kind of secreted glycoprotein and is involved in maintaining normal structure and function of skin. Ultraviolet exposure significantly up‐regulates its expression, which may disrupt dermal homeostasis and enable abnormal differentiation of keratinocytes, ultimately leading to photoaging (Hardy et al., 2019).

Our study also revealed three possible anti‐aging targets. Telomere plays a vital role for maintaining genomic stability, and telomere attrition is connected with aging. MAX were negatively associated with TL and could activate telomerase gene promoter to prevent telomere shortening (Zhang et al., 2020).

Even though we compared the MR results from three different cohorts, our study also has several limitations. First of all, it is difficult to completely prevent residual bias in MR studies despite the fact that we conducted multiple methods to reduce confusion resulted from pleiotropy. Besides, due to the lack of data, we only included several aging proxy indicators and ignored other aging markers, such as epigenetic changes, mitochondria malfunction, senescence‐associated secretory phenotype (SASP), and so on, which leads to the omission of potential associations (Bao et al., 2023). Third, data of three cohorts (Decode, FinnGen and validation) were all derived from Caucasians. Therefore, our results may have bias owing to population heterogeneity, which requires us to include GWASs from different populations and races in the future. Finally, it should be acknowledged that RCTs have stronger causality than MR analysis. In that case, more clinical trials with high quality are needed to support our results.

The present study found 10 proteins which may affect aging. Furthermore, we also performed phenome‐wide analysis of these candidate proteins. Additionally, we found that the moderation effect proteins had on aging could be mediated by metabolites like 1,5‐anhydroglucitol. Collectively, our results provided several potential targets for delaying aging.

## AUTHOR CONTRIBUTIONS

RM and WX wrote an original draft, software, investigation, visualization, methodology, and conceptualization. JL performed software, data source, and methodology. WX was responsible for supervision, conceptualization, writing—original draft, and funding acquisition.

## FUNDING INFORMATION

This research was generously supported by the National Natural Science Foundation of China (grants: 82273557, 82373508, 82003385, 82173448, 82203958, 82203945, 82073457), the National Natural Science Funds for Distinguished Young Scholars (grant: 82225039), National Key Research and Development Program of China (grant: 2023YFC2509003) and Natural Science Foundation of Hunan Province, China (grant: 2024JJ6680).

## CONFLICT OF INTEREST STATEMENT

The authors declare no conflicts of interest.

## Supporting information


Appendix S1

Appendix S2

Appendix S3
