# Supplementary material for: Identification of prospective aging drug targets via Mendelian randomization analysis
Source: Aging Cell. 2024 Apr 4;23(7):e14171. doi: 10.1111/acel.14171 (PMC11258487; doi:10.1111/acel.14171)
Supplement: Supplementary file 2 — Appendix S2 [file ACEL-23-e14171-s001.docx]

**Supplement 2**

**eFigure 1**. Conceptual Framework for Mendelian Randomization and Its Core Assumptions.

**eFigure 2:** Process of Mendelian Randomization analysis for identifying potential drug targets of longevity across three distinct cohorts.

**eFigure 3:** Phenome-Wide Mendelian Randomization (MR) Manhattan Plot Displaying the Consequences of Blood Protein Expressions.

**eFigure 4:** Phenome-Wide Mendelian Randomization (MR) Manhattan Plot Demonstrating the Consequences of Blood Protein Expressions


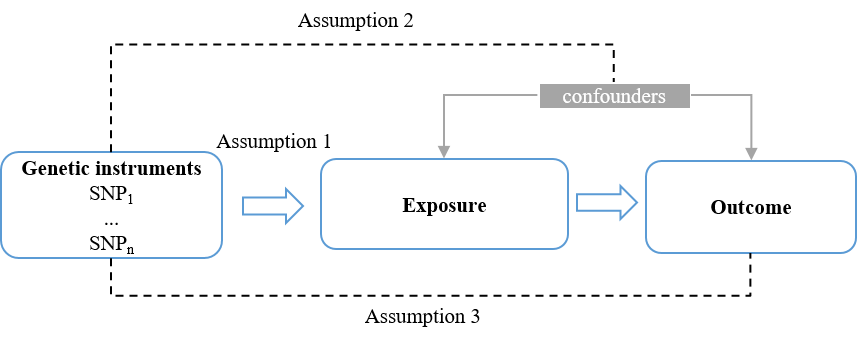


**eFigure 1**. Conceptual Framework for Mendelian Randomization and Its Core Assumptions.

Assumption 1: The genetic variant must exhibit a robust association with the intended exposure. Assumption 2:The instrumental genetic variants should operate independently of any confounders that could affect the exposure-outcome nexus. Assumption 3: Genetic variants should exert influence on the outcome exclusively through their effect on the designated exposure.


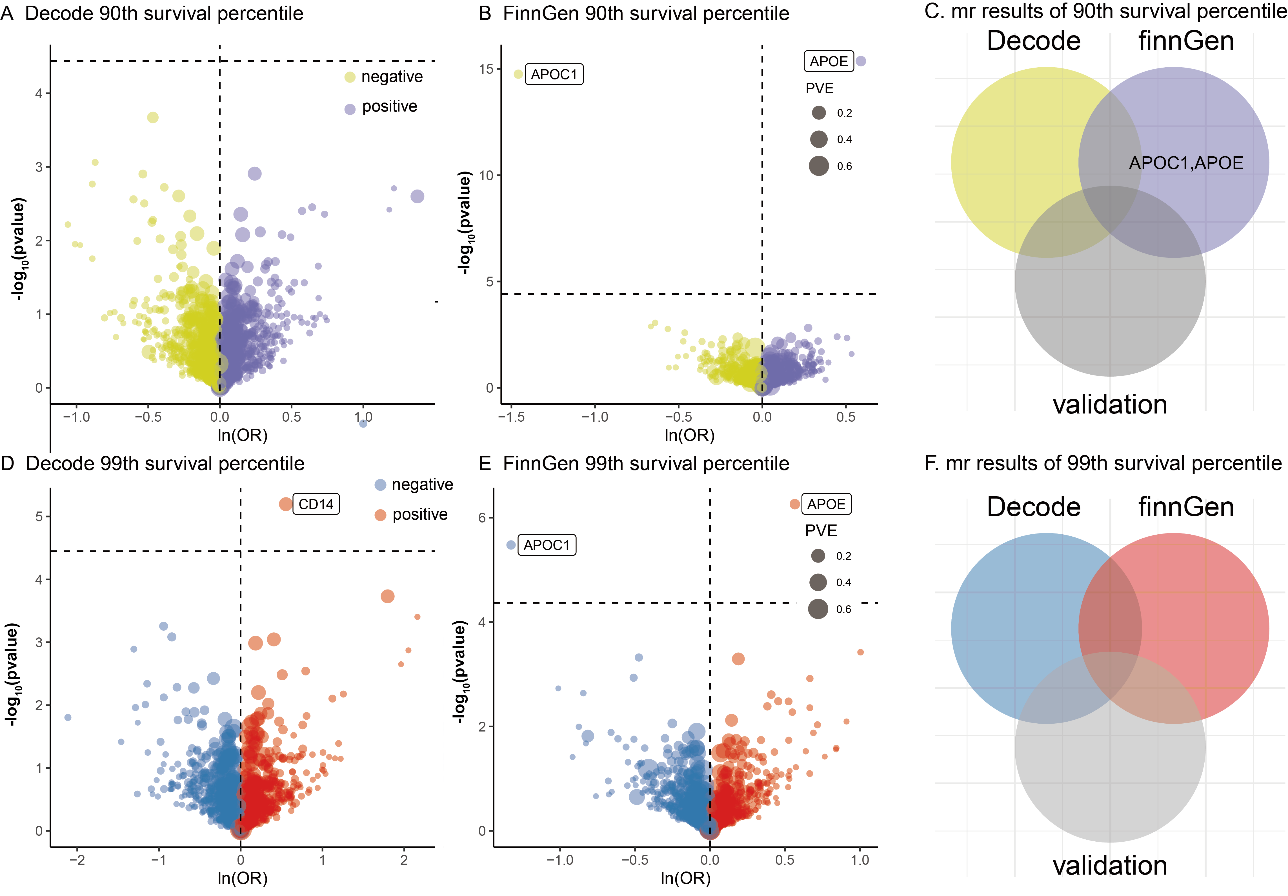


**eFigure 2: Process of Mendelian Randomization (MR) analysis for identifying potential drug targets of longevity across three distinct cohorts.**

(A) Volcanogram illustrating the risk association between plasma proteins of the DECODE cohort and 90th percentile age. A delineated horizontal black line signifies P = 3.65 × 10^-5^ (0.05/1369).

(B) Volcanogram representing the risk association of plasma proteins from the Finnland cohort with 90th percentile age, marked by a dashed horizontal black line at P = 3.77 × 10^-5^ (0.05/1323).

(C) Comparative analysis intersecting drug targets for 90th percentile age from the three cohorts.

(D) Volcanogram showcasing the risk association between plasma proteins from the DECODE cohort and 99th percentile age, indicated by a delineated horizontal black line at P = 3.68 × 10^-5^ (0.05/1358).

(E) Volcanogram highlighting the risk association of plasma proteins from the Finnland cohort with 99th percentile age, with a dashed horizontal black line at P = 3.81 × 10^-5^ (0.05/1312).

(F) Comparative intersection of drug targets for 99th percentile age sourced from the three cohorts.

The odds ratio (OR) for increased psoriasis risk is depicted as a standard deviation (SD) increment for each rise in plasma protein level. Key: ln = natural logarithm; PVE = proportion of variance explained.


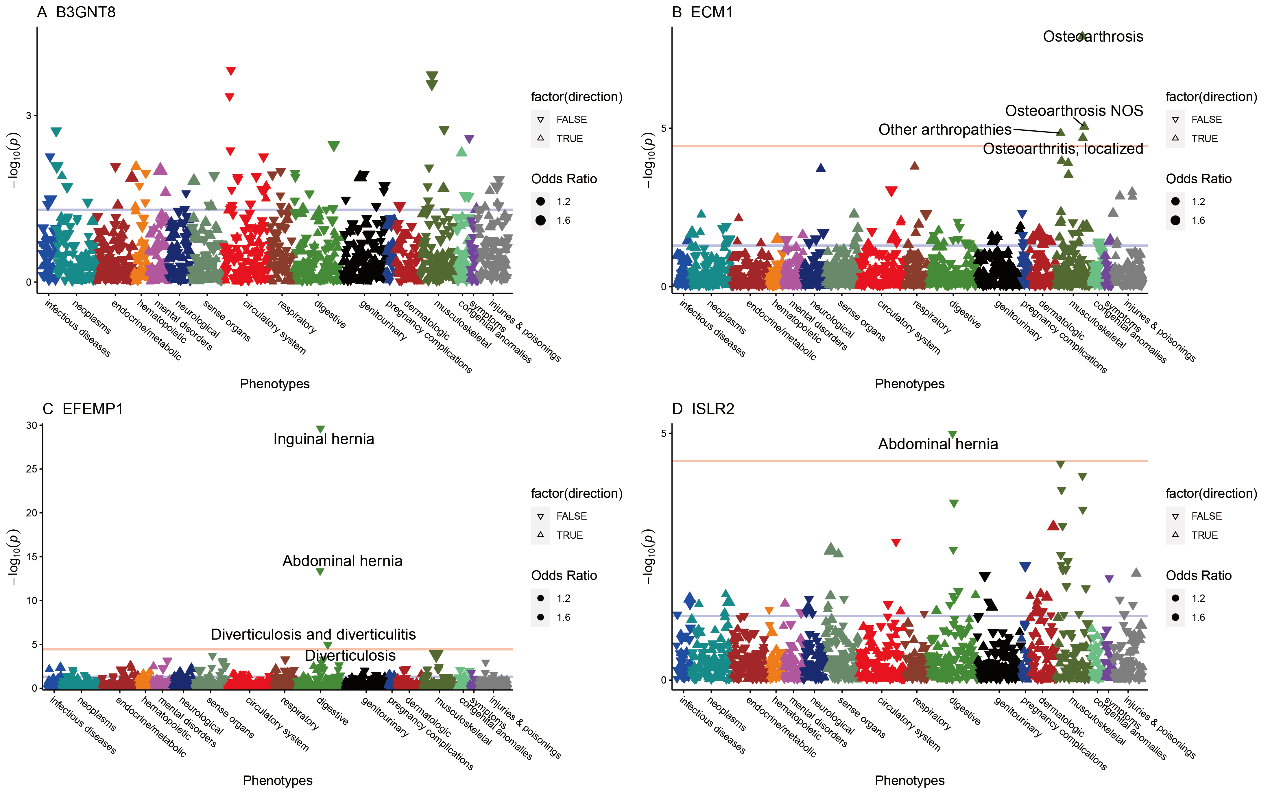


**eFigure 3:** Phenome-Wide Mendelian Randomization (MR) Manhattan Plot Displaying the Consequences of Blood Protein Expressions: B3GNT8 (A), ECM1 (B), EFEMP1 (C), and ISLR2 (D) on a Spectrum of 1,402 Diseases, as cataloged in the UK Biobank (UKB). It's pivotal to note that the ordinate showcases the P-value derived from the phenome-wide MR analysis. A gray demarcation line corresponds to a P-value threshold of 0.05. In contrast, a pink line represents an adjusted significance threshold of 3.56e^-5^ (0.05/1402). Each triangle icon denotes a specific disease. Upward-facing triangles intimate a positive causal influence of the blood protein, while downward-oriented triangles suggest an inverse causal association.


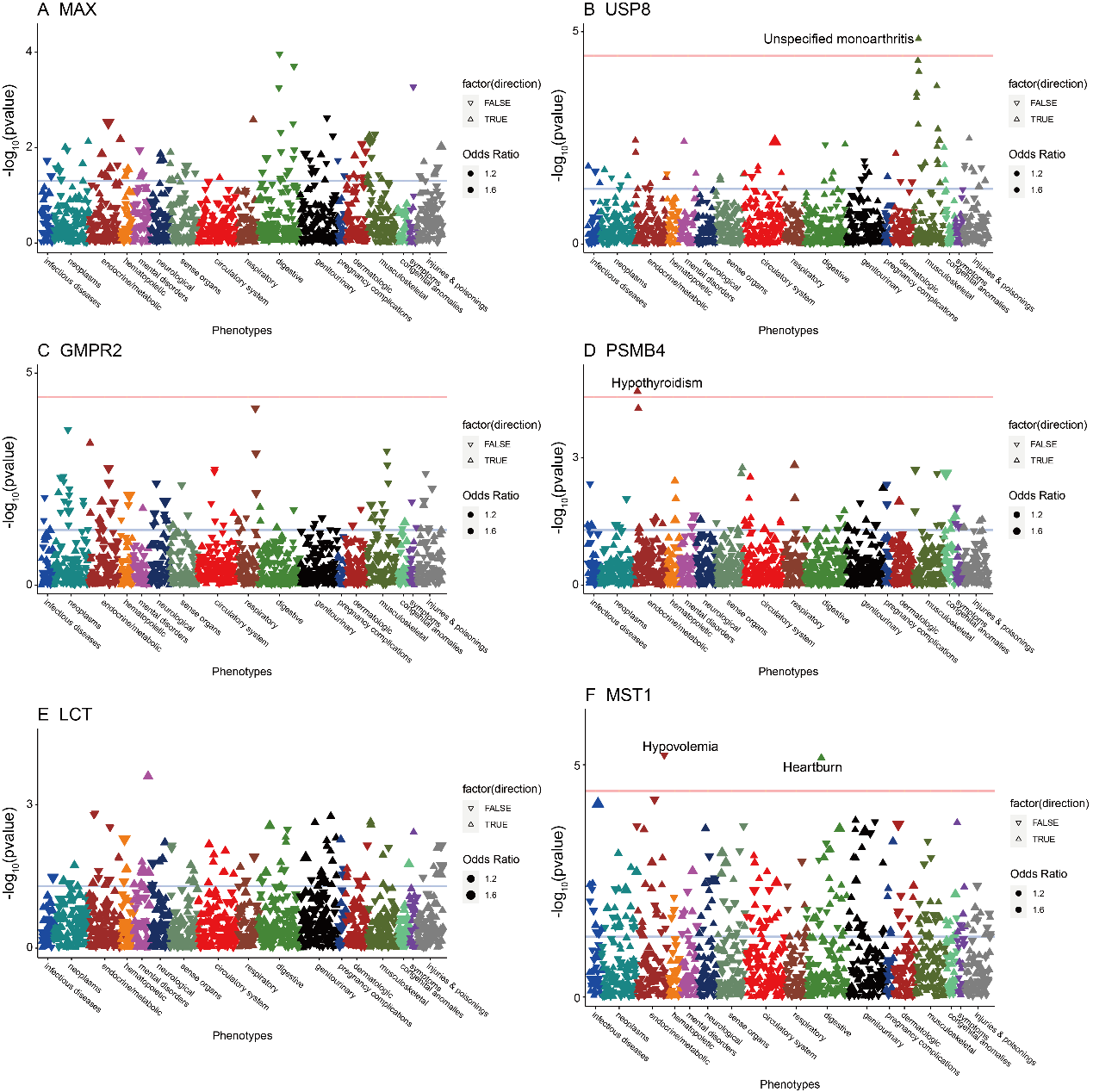


**eFigure 4:** Phenome-Wide Mendelian Randomization (MR) Manhattan Plot Demonstrating the Consequences of Blood Protein Expressions for LCT (A), GMPR2 (B), MAX (C), USP8 (D), PSMB4 (E), and MST (F) across a compilation of 1,402 Diseases, as cataloged within the UK Biobank (UKB). Notably, the ordinate depicts the P-value derived from the phenome-wide MR analysis. A distinct gray line establishes a P-value threshold of 0.05, while a contrasting pink line represents the adjusted significance level of 3.56e^-5^ (0.05/1402). Each triangular marker represents a specific disease, with upward-facing triangles indicating a positive causal influence of the blood protein and downward-oriented triangles suggesting a contrary causal association.
