# Supplementary material for: Identification of prospective aging drug targets via Mendelian randomization analysis
Source: Aging Cell. 2024 Apr 4;23(7):e14171. doi: 10.1111/acel.14171 (PMC11258487; doi:10.1111/acel.14171)
Supplement: Supplementary file 3 — Appendix S3 [file ACEL-23-e14171-s003.docx]

| **Support table 1. Associations Between Genetically-Proxied Protein Expression and Aging-Related Traits (Using UKB-PPP cohort as Exposure).** | | | | | | | | | | | | |
| --- | --- | --- | --- | --- | --- | --- | --- | --- | --- | --- | --- | --- |
| **outcome** | **exposure** | **method** | **nsnp** | **b** | **se** | **pval** | **pve** | **lo_ci** | **up_ci** | **or** | **or_lci95** | **or_uci95** |
| telomere length | GMPR2 | Wald ratio | 1 | -0.03318 | 0.016745 | 0.047539 | 0.007467 | -0.066 | -0.00036 | 0.967364 | 0.93613 | 0.999641 |
| telomere length | MAX | Wald ratio | 1 | 0.20008 | 0.026021 | 1.48E-14 | 0.002946 | 0.149079 | 0.251081 | 1.221501 | 1.160765 | 1.285414 |
| telomere length | USP8 | Wald ratio | 1 | 0.137439 | 0.027478 | 5.68E-07 | 0.002674 | 0.083582 | 0.191296 | 1.147332 | 1.087175 | 1.210818 |
| facial aging | ECM1 | Wald ratio | 1 | 0.028874 | 0.006531 | 9.84E-06 | 0.177542 | 0.016072 | 0.041676 | 1.029295 | 1.016202 | 1.042556 |
| facial aging | EFEMP1 | Wald ratio | 1 | 0.132263 | 0.02284 | 7.01E-09 | 0.021435 | 8.75E-02 | 0.17703 | 1.141408 | 1.091438 | 1.193666 |
| facial aging | ISLR2 | Wald ratio | 1 | 0.067068 | 0.021243 | 0.001593 | 0.023769 | 0.025432 | 0.108705 | 1.069369 | 1.025758 | 1.114834 |
| frailty index | MST1 | Wald ratio | 1 | 0.0178 | 0.003427 | 2.05E-07 | 0.309301 | 0.011083 | 0.024516 | 1.017959 | 1.011145 | 1.024819 |
